# Effectiveness of the secondary distribution of HIV self-testing with and without monetary incentives among men who have sex with men living with HIV in China: study protocol for a randomized controlled trial

**DOI:** 10.1186/s12879-023-08062-w

**Published:** 2023-03-14

**Authors:** Siyue Hu, Ying Lu, Xi He, Yi Zhou, Dan Wu, Joseph D. Tucker, Bin Yang, Weiming Tang

**Affiliations:** 1 School of Public Health, Southern Medical University, Guangzhou, China; 2 Dermatology Hospital of Southern Medical University, Guangzhou, China; 3 University of North Carolina Project – China, Guangzhou, China; 4 Zhuhai Xutong Voluntary Services Center, Zhuhai, China; 5 Zhuhai Center for Diseases Control and Prevention, Zhuhai, China; 6 London School of Hygiene and Tropical Medicine, London, UK; 7 West China School of Public Health, Sichuan University, Chengdu, China

**Keywords:** HIV self-testing, People living with HIV, Men who have sex with men, Monetary incentives, Secondary distribution

## Abstract

**Background:**

The HIV epidemic is still expanding among men who have sex with men (MSM) in China, but HIV testing rates remain suboptimal. Network-based interventions, such as secondary distribution, have shown promise to expand HIV self-testing (HIVST) among partners of MSM living with HIV (MLWH) but have not been widely implemented. Monetary incentives could enhance the secondary distribution of HIVST in some settings. We will conduct a randomized controlled trial to examine the effectiveness of monetary incentives in expanding the secondary distribution of HIVST among MLWH in China.

**Methods:**

We will recruit 200 eligible participants at three antiretroviral therapy (ART) clinics in China. Participants are eligible if they are 18 years of age or over, assigned as male at birth, have had anal sex with men, are living with HIV, are willing to apply for the HIVST kit at ART clinics, and are willing to provide personal contact information for follow-up. Eligible participants will be randomly assigned in a 1:1 ratio to one of two groups: standard secondary distribution group and secondary distribution group with monetary incentives. Participants (defined as “index”) will distribute the HIVST kits to members of their social network (defined as “alter”) and will be required to complete a baseline survey and a 3-month follow-up survey. All alters will be encouraged to report their testing results by taking photos of used kits and completing an online survey. The primary study outcomes will compare the mean number of alters and newly-tested alters motivated by each index participant in each group. Secondary study outcomes will include the mean number of alters who tested positive, the cost per person tested, and the cost per HIV diagnosed for each group.

**Discussion:**

Few studies have evaluated interventions to enhance the implementation of secondary distribution. Our study will provide information on the effectiveness of monetary incentives in expanding HIVST secondary distribution among MLWH. The findings of this trial will contribute to implementing HIVST secondary distribution services among MLWH in China and facilitating HIV case identifications.

**Trial registration:**

Chinese Clinical Trial Registry ChiCTR2200064517; http://www.chictr.org.cn/showproj.aspx?proj=177896. Registered on 10th October 2022.

## Background

Key populations account for less than 5% of the global population, but they and their sexual partners comprised 70% of new HIV infections in 2021 [1]. In China, the HIV prevalence among MSM was 5.4% by 2021, but only 59% of MSM knew their HIV status [2]. Although traditional facility-based HIV testing services have expanded HIV testing among MSM in China, additional strategies are still needed to improve HIV testing coverage among MSM [3–6].

HIV self-testing (HIVST) complements traditional facility-based HIV testing services and has been recommended by the World Health Organization (WHO) and other HIV testing service guidelines [7, 8]. HIVST has participants collect their own samples, perform the test, and read the results themselves [9]. It is a convenient, rapid, and discrete HIV testing strategy. Moreover, HIVST can identify and link additional people with HIV to care, effectively improving the detection rate and expanding HIV testing coverage in MSM [10, 11]. Therefore, HIVST is gradually being promoted as an HIV testing strategy to expand HIV testing services for key populations. Among the HIVST distribution strategies, secondary distribution is an innovative method that is one of the most promising in promoting HIV testing among people in the social network of the index key populations [12, 13]. Studies have shown that this model has the potential to be a new measure of HIV prevention and control in MSM populations, serving the dual purpose of promoting first HIV testing and detecting positive infections [14–16].

People living with HIV (PLWH) are vital in ongoing HIV transmission, and their behavioral characteristics determine the rate and extent of HIV transmission [17]. MSM have large sexual networks, and there is evidence that the proportion of MLWH infected by casual or regular partners has been increasing in recent decades [18]. HIVST may help to expand HIV testing services among sexual partners of MLWH. PLWH can serve as a source of intervention by encouraging their sexual partners to get tested. A pilot study conducted in Vietnam showed that it is feasible and effective to facilitate HIV testing by assisting PLWH in contacting their sexual partners and providing free HIVST kits [19].

Secondary distribution of HIVST kits among social networks of MLWH, especially among sexual partners, may improve HIV testing coverage. However, some barriers remain. Psychological and behavioral economic theories suggest that individuals prefer the “present”: over-valuing the present and severely under-valuing the future [20, 21]. This implies that information alone may not be sufficient to change behavior when there are direct costs and delayed benefits to a behavior. As in the case of HIVST, there are direct logistical and psychological costs, while the benefits of HIVST become apparent later [22]. Using monetary incentives can increase direct benefits, offset current costs, and spur behavior change. There is growing evidence that monetary incentives, particularly fixed monetary incentives and conditional cash transfers, increase the demand for and acceptance of HIV testing [23–26]. Moreover, few studies have explored the effects of monetary incentives to use HIVST among sexual partners in MLWH. Further research is urgently needed to fill the current gap, which could also be used to complement studies exploring the cost or cost-effectiveness of secondary distribution strategies in PLWH.

In this study, we propose to explore the effectiveness of the secondary distribution of HIVST with and without monetary incentives among MLWH through a randomized controlled trial in Zhuhai, China.

### Objectives

The primary objective of our trial is to evaluate the effectiveness of the monetary incentives in expanding the secondary distribution of HIVST among MLWH.

### Hypotheses

We hypothesized secondary distribution with monetary incentives would be more effective in expanding the secondary distribution of HIVST among MLWH and would identify more MSM living with HIV but not aware of their infection status.

## Methods/design

### Trial design

**Fig. 1:**
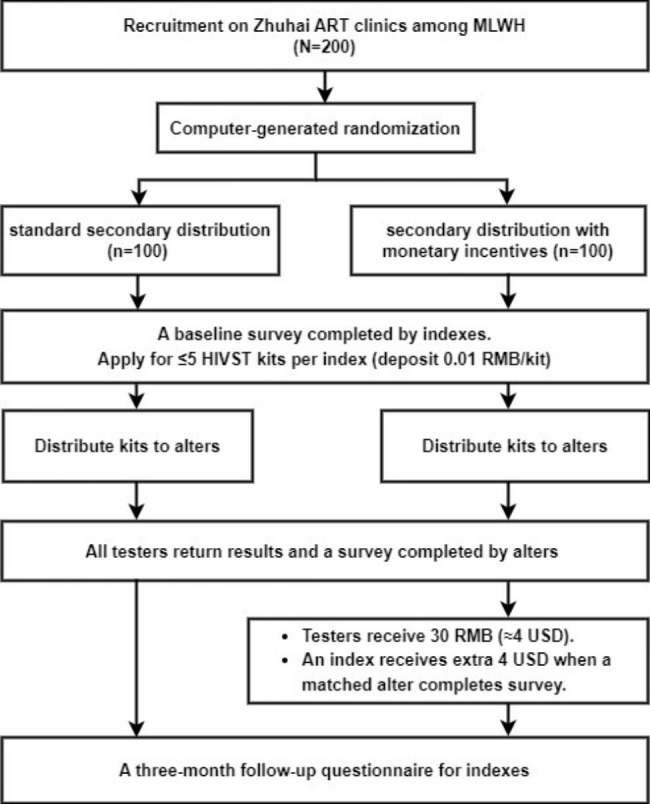
Trial Flowchart

The study is a randomized controlled trial in which MLWH who meet the recruitment criteria are randomized into two groups: standard secondary distribution group and secondary distribution with monetary incentives group. All enrolled MLWH will be offered several HIVST kits in ART clinics. Participants will complete the baseline survey at the start of the trial and the corresponding alter will complete the online survey when the HIVST results are returned (Fig. 1).

### Study setting and recruitment

Building on existing research [16, 27], the Social Entrepreneurship to Spur Health (SESH) research team, the Zhuhai Center for Disease Control and Prevention (CDC), and the Zhuhai Xutong Volunteer Service Center (Xutong), an MSM-friendly community-based organization (CBO), will further strengthen their collaboration to pinpoint the target population from MSM to MLWH. The MLWH will be recruited in Zhuhai through an online ordering system developed by Xutong, which is managed on WeChat, the largest social networking platform in China.

The study will be conducted at three ART clinics in Zhuhai: two designated hospital clinics of Zhuhai CDC (The Fifth Affiliated Hospital of Sun Yat-sen University and The Fifth Affiliated Hospital of Zunyi Medical University) and Xutong community health service station (Lingnan Community Health Service Station). These clinics and their services are selected for the study because they are developed with input for MSM and provide “one-stop” services. More specifically, the “one-stop” service is a platform for HIV-screening providers to give all service referrals for screen-positive patients at designated ART hospitals, which offer specialized medical services, including consultation, epidemiological investigation, follow-up, HIV confirmatory sample delivery, and antiretroviral therapy. All clinics are staffed by MSM peer volunteers, nurses, and public health staff. Study recruitment will be conducted face-to-face with MLWH at these three sites, where researchers will fully introduce the specifics of the project to MLWH and invite them to participate in the study after a scientific introduction on how to use the HIVST kits by themselves.

### Eligibility criteria

Participants are eligible for inclusion only if they met all of the following inclusion criteria: (1) be assigned male gender at birth; (2) at least 18 years or older; (3) ever had anal sex with men; (4) living with HIV; (5) willing to apply for HIVST kits at ART clinics; (6) willing to provide personal contact information for follow-up.

### Randomization and allocation

A biostatistician who is not involved in participant enrollment will produce and keep computer-generated randomization codes. Eligible MLWH participants will be electronically randomized individually and independently to the intervention and control groups through a computer-generated procedure based exclusively on a 1:1 allocation ratio. At the site, index participants will be asked to randomly select a random code via a computer-generated electronic program after the baseline survey. The researcher will group participants according to the random code.

### Interventions

We will randomly assign participants to either the standard secondary distribution group as the control group or the secondary distribution with the monetary incentives group as the intervention group. All enrolled MLWH will receive HIV self-test kits at the ART clinics.

### Standard secondary distribution group/ control group

MLWH assigned to this group will serve as the index participants in this group for the secondary distribution of HIVST. They will complete a baseline survey and apply for up to 5 HIVST kits as needed, paying a deposit based on the number of HIVST kits claimed (0.01RMB deposit per kit). Setting up a deposit is to verify the WeChat account, thus facilitating the issuance of subsequent incentives and obtaining contact information for follow-up visits to ensure results return. Each HIVST kit will be packaged in an unmarked box to protect privacy, in addition to the HIVST instructions and a “return card” included inside the box. The return card contains a QR code that allows testers to anonymously and privately scan and upload a photo of their test results to the online platform. The return card also contains a confirmation number to identify the different index participants and their corresponding alters. After receiving the HIVST kit, MLWH index participants will distribute it to their sexual partners, friends, or others in their social network. The alters accepting the self-test kits and returning the results will be considered a successful distribution.

### Secondary distribution with monetary incentives group/ intervention group

MLWH in this group will follow the same process as the control group. The difference is that when an alter accepts the HIVST kit and returns the result, both the index who distributed to the alter and the alter himself/herself will receive a bonus reward of 30 RMB (≈ 4 USD) as monetary incentives. All incentives will be provided through online trading. Within this group, index participants can distribute up to 5 alters and obtain incentives of up to 150 RMB.

### Consultation, follow-up, and referral services

Research staff will provide face-to-face counseling or online counseling before and after the test. On-site, trained research staff, will provide pre-test consultation to all participants and fully inform them how to use HIVST kits in person when they apply for a test kit. In addition, each HIVST kit will also include instructions. Post-test consultation for those who upload HIV-negative results will include a reminder of what the test result means and how to prevent HIV infection. Individuals with “positive” or “inconclusive” test results will be contacted immediately via phone or WeChat. Individuals with positive self-test results will be offered post-test consultation services, including a detailed explanation of test results and timely referrals to clinics for confirmatory tests. In addition, researchers will encourage positive testers to pass on the HIVST kit to their sexual partners or provide contact information for their sexual partners, who researchers will contact to provide free testing services.

### Study measures

For the baseline survey, index participants who meet the inclusion criteria will complete the baseline questionnaire. We will collect socio-demographic characteristics (age, gender, marital status, the highest level of education completed, and monthly income), sexual behavior (previous sex, role during sex with men, condom use, number of sexual partners, and drug use), influence within personal social circles, previous HIV testing experience and their social network.

After three months, we will follow up with the index MLWH, asking them about the distribution process, focusing on their experience and attitude in distributing the self-test kits and their willingness to recommend them. In addition, when alters upload their results, they will also be required to complete a questionnaire online. We will ask about their socio-demographic characteristics, sexual behavior, the experience of previous HIV testing, experiences and attitudes toward using HIVST kits, and their social network.

Participants are required to collect their fingertip blood samples according to the instructions for use. All blood samples will be analyzed using the SD Bioline HIV/Syphilis duo test kits (SD Bioline, Company, South Korea). The trained staff at CBO will examine the resulting photographs and provide corresponding post-test consultation, which will be recorded in the JINSHUJU.

### Outcomes

Our primary outcome will be (1) the mean number of alters motivated by each index in each group, which will be compared between the two groups; (2) the mean number of newly tested alters motivated by each index in each group. Secondary outcomes will be (1) the mean number of alters who tested positive for HIV recruited by index participants in each group; (2) the cost per person tested and HIV diagnosed for each group.

### Sample size

Based on the results of the pilot study and related studies, the number of alters motivated by the index through standard secondary distribution was set at 0.65, and through the secondary distribution with monetary incentives, was set at 1.0, assuming equal variance between the two groups, we used a standard deviation of 0.5 for all groups [16, 27]. We set the required sample size at 100 people, for a total of 200 people, with an alpha of 0.05, a power of 0.90, and a lost follow-up rate of 0.20.

### Data management

All data will be stored electronically at JINSHUJU, a secure online platform that records HIVST kit application information, questionnaire information, and uploaded test results. A contractual agreement with JINSHUJU and JINSHUJU’s encryption system guarantee the privacy of the data. Only project researchers have access to the JINSHUJU, thus ensuring the security and privacy of participants. When transferring data, some identifiable variables (such as phone numbers and addresses) will be encrypted to protect privacy. During the trial, participants will be contacted on a daily basis using a specific phone for telephone support and post-test consultation.

### Missing data plan

Index participants will be involved in the baseline survey and the subsequent three-month follow-up survey, and alter participants will be involved in the online questionnaire phase when tests results are uploaded. However, for our primary and secondary outcomes, data may be missing. For participants missing at follow-up, we will document the reason for missing data and use suitable imputation to improve the robustness of the study results.

### Data analysis

All statistical analyses will follow the intention-to-treat principle. We will use descriptive analysis to report baseline characteristics. For baseline differences between index participants who completed the 3-month follow-up survey and those who did not, we will use a two-sample t-test for comparison if the data are normally distributed and a chi-square test if not. A p-value less than 0.05 is considered statistically significant.

Considering the distribution of the results and based on related studies, we will use zero-inflated negative binomial regression for the primary outcome analysis [27, 28]. The primary outcome analysis will compare the differences between the control and intervention groups. Then the incidence rate ratio (IRR) and its associated 95% CI will be estimated further to compare the primary outcome difference between the two groups. For the secondary outcome analysis, we will compare differences in the mean number of alters who tested positive for HIV recruited by index participants in each group and further assessed by IRR and its associated 95% CI. In addition, we will also apply logistic regression for the secondary outcome analysis. We will perform a subgroup analysis of the first primary outcome, stratified by statistically significant factors. All IRRs will be adjusted by index participant-related factors.

If possible, we will conduct an economic evaluation by using micro-costs to assess the total cost per group, the cost per person tested and the cost per HIV diagnosed for each group. Also, we will compare the cost-effectiveness in the two groups, intervention and control groups.

## Discussion

Enhancing HIV testing services among MSM is essential for HIV control [29, 30]. Secondary distribution of HIVST based on social networks of MSM is feasible and contributes to the uptake of HIV testing in Chinese MSM [16, 27]. However, few studies have evaluated interventions directly imposed on the social networks of PLWH to increase awareness and prevention of HIV. This study will extend the existing literature by implementing secondary distribution of HIVST among MLWH only, promoting partner testing and friends testing, and assessing whether monetary incentives can improve its effectiveness.

Several limitations should be considered in this study. First, even if the self-test kits are free, the deposit set for each kit may discourage order and distribution by participants who may have to cover the deposit expenses for their peers. This study has minimized the deposit threshold to a deposit amount of 0.01 RMB per kit, which is affordable for everyone to ensure that the deposit does not become a barrier for participants to apply for self-test kits. Second, due to the three-month interval between follow-up surveys, follow-up data may be lost during the information collection process resulting in biased results. As compensation, the researchers will evaluate whether some of the participants’ questionnaire variables are consistent before and after follow-up. Third, participants may migrate if the study period is long. To avoid this situation, we will closely track participants and remind them to contact us promptly to update their phone numbers or WeChat. Finally, from an implementation and outreach perspective, although each participant can apply for HIVST kits multiple times, there is only a maximum of five requests per person, which may not meet the needs of participants willing to distribute as many self-test kits as possible. However, we can address this issue by removing the limit in the future implementation study.

New strategies for more targeted and effective testing are needed to sustain HIV services during the COVID-19 pandemic. HIVST does not require direct contact interaction, which can meet the unique planning needs and global HIV testing goals during this period and over the long term [31]. In addition, the secondary distribution of social network-based allows marginalized populations to receive free HIV self-testing at a very low threshold, which their friends turn in. It will reach marginalized, high-risk MSM hidden in current HIV testing practices and encourage intra-network conversations about risk behaviors, HIV testing, and HIV status, further promoting accurate HIV intervention and control. A randomized controlled trial showed that monetary incentives effectively promoted the secondary distribution of HIVST and expanded HIV testing among MSM [27]. Building on this, our study will further narrow the target population to focus on PLWH, thus further validating whether it can more efficiently identify new HIV-positive cases and facilitate ART initiation. Also, we will compare the cost of secondary distribution of HIVST in two populations, MSM and MLWH, to further complement studies exploring the cost-effectiveness of secondary distribution strategies in PLWH.

We will use innovative strategies based on the HIVST secondary distribution model to improve case-identifying management efficiency and target social networks of MLWH for accurate interventions with the help of the one-stop service platform. We aim to promote HIV testing in the social networks of MLWH, expand testing in high-risk populations, cut off high-risk HIV transmission pathways, and explore more effective secondary distribution models. This will provide an important theoretical basis for further promoting the application of these intervention technologies in other populations and other regions.

### Trial status

We have designed a study schedule from October 2022 to October 2023. The recruitment process and data collection are ongoing at the time of writing this draft protocol. We will close the recruitment system on July 31, 2023, and expect to complete all follow-up work by October 2023. Statistical analysis has not yet begun.

## Data Availability

Data sharing is not applicable to this article as no datasets were generated or analysed during the current study.

## Abbreviations

ART: Antiretroviral Therapy
CBO: Community-Based Organizations
CDC: Center for Disease Control and Prevention
CI: Confidence interval
HIV: Human Immunodeficiency Virus
HIVST: HIV Self-Testing
MSM: Men Who Have Sex with Men
MLWH: MSM Who are living with HIV
PLWH: People living with HIV
QR: Quick response (barcode)
SD: Secondary Distribution
SESH: Social Entrepreneurship to Spur Health Group
Xutong: Zhuhai Xutong Voluntary Services Center
WHO: World Health Organization
